# Heterogeneity characterization and key pathogenic genes screening based on diabetic nephropathy microenvironment

**DOI:** 10.1515/biol-2025-1273

**Published:** 2026-04-17

**Authors:** Miao Tan, Jingjing Xue, Jinchuan Tan, Meifang Ren, Qian Zhang, Suzhi Chen, Ruijing Song, Yuhan Nui, Yicong Zhao, Yongzhang Li, Fengwen Yang

**Affiliations:** Department of Endocrinology, The Fourth Hospital of Hebei Medical University, 12 Jiankang Road, Shijiazhuang, Hebei Province 050011, China; Department of Nephrology, Hebei University of Traditional Chinese Medicine, 3 Xingyuan Road, Shijiazhuang, Hebei Province 050200, China; The First Department of Nephrology, Hebei Provincial Hospital of Traditional Chinese Medicine, 389 Zhongshan East Road, Shijiazhuang, Hebei Province 050017, China; Research Center, Hebei Provincial Hospital of Traditional Chinese Medicine, 389 Zhongshan East Road, Shijiazhuang, Hebei Province 050017, China

**Keywords:** diabetic nephropathy, microenvironment, consensus clustering, heterogeneity differentially expressed genes

## Abstract

The inflammatory response is a direct factor leading to changes in the microenvironment of renal tissues. The immune cells and stromal cells infiltration were the essential characteristics of diabetic nephropathy (DN). Based on the differentiation of the microenvironment, describing the heterogeneity of DN may provide a new approach to explore the mechanisms of disease progression. This study was aimed to classify DN samples based on the infiltration levels of immune cells and stromal cells, describe the microenvironment heterogeneity of DN samples, explore the potential mechanisms of phenotypic differentiation, screen key pathogenic genes, construct a quantitative scoring model to describe the microenvironment, and investigate the role of key pathogenic genes of DN. We downloaded RNA sequencing datasets of DN tissue and normal kidney tissue (GSE142025 and GSE96804) from the Gene Expression Omnibus (GEO) database. The RNA sequencing data was transformed into immune cell and stromal cell infiltration data using the xCell algorithm. Ward’s method was used for consensus clustering to identify different phenotypes of DN. We screened out the key pathogenic genes associated with phenotypes, and established a scoring model through principal component analysis which was tested the reliability and accuracy in the training cohort and the validation cohort. We explored the influence of key pathogenic genes on the biological behavior of human mesangial cells. Based on the heterogeneity of the diabetic nephropathy (DN) microenvironment, this study identified 15 key pathogenic genes: FN1, EGR1, TPM1, CCND2, COL1A2, TGFB2, COL6A3, ITGA11, ABCC9, THBS2, TNC, COL3A1, C7, C1QC, and ITGB6. The PCA score constructed from these genes (i.e., their first principal component, PC1) demonstrated good efficacy in distinguishing normal samples from DN samples (AUC = 0.90, 95 % CI [0.82–0.97]), differentiating DN subtypes with distinct microenvironments (AUC = 0.99, 95 % CI [0.97–1.00]), and stratifying DN samples at different disease stages. This score showed significant positive correlations with immune score (r = 0.75, *P* = 1.6e-7) and stromal score (r = 0.96, *P* < 2.2e-16). Under high-glucose stimulation, the protein and mRNA expression of ABCC9 in mesangial cells increased over time. Knockdown of ABCC9 partially counteracted the high glucose-induced increase in apoptosis and enhancement of migration in mesangial cells. In an external validation cohort, both the PCA score (AUC = 0.91 for distinguishing normal from DN; AUC = 0.95 for differentiating DN subtypes) and ABCC9 expression (AUC = 0.93 for distinguishing normal from DN; AUC = 0.90 for distinguishing early from advanced DN) further confirmed their association with the DN microenvironment and disease progression. FN1, EGR1, TPM1, CCND2, COL1A2, TGFB2, COL6A3, ITGA11, ABCC9, THBS2, TNC, COL3A1, C7, C1QC, and ITGB6 are potential key pathogenic genes in DN. The PCA score constructed based on these genes can distinguish between normal tissue phenotype and DN phenotype, quantitatively describing the immune microenvironment and stromal microenvironment of DN, reflecting the progression of DN. Knocking out ABCC9 can counteract the increased apoptosis and migration of glomeruli mesangial cells induced by high glucose.

## 1 Introduction

Diabetic nephropathy (DN) is characterized by albuminuria and/or decreased glomeruli filtration rate [1]. It is worth noting that even mild albuminuria or a slight decrease in GFR can significantly increase the risk of advanced kidney disease and premature death in patients with D [2], 3]. The incidence and prevalence of DN have significantly increased in the past decade [4]. Compared to common clinical glomeruli diseases such as membranous nephropathy, DN progresses rapidly, and many patients inevitably progress to ESRD, relying on dialysis treatment to maintain basic survival [5], 6]. The onset of the disease is often insidious, and many patients are already in the mid-stage of the disease when diagnosed. Currently, the pathogenesis of the disease is not clear, and clinical treatment has not achieved satisfactory results [7], 8]. DN poses a serious threat to human health and quality of life, and in-depth study of its pathogenesis is with importance in the early prevention, diagnosis, and treatment of DN [9].

For a long time, the hyperglycemic status has been regarded as the initiation factor of diabetic complications including DN [10]. Abnormal glucose and lipid metabolism and renal hemodynamic disorders caused by high blood glucose are the two major pathological bases of DN [11]. Based on this, the current treatment of DN mainly focuses on controlling blood glucose, blood lipids, blood pressure, and improving renal hemodynamics [12], 13]. However, comprehensive management that achieves target levels of blood glucose, blood lipids, blood pressure, etc. cannot completely prevent the occurrence and development of DN, suggesting the involvement of other pathological processes in the onset and progression of DN [14], 15]. In DN patients, lymphocytes, macrophages, and hypertrophic cells accumulate in renal tissues, which secrete inflammatory mediators, cytokines, and reactive oxygen species, directly or indirectly inducing damage to renal tissues and accelerating the process of renal fibrosis [16], [17], [18]. Metabolic disorders in DN patients activate inflammatory signals in the body, leading to the deposition of extracellular stromal in the kidneys and promoting fibrosis. Therefore, inflammation is one of the important links in the continuous progression of diabetic complications including DN [19]. The inflammatory response is a direct factor leading to changes in the microenvironment of renal tissues [20], 21]. The immune cells and stromal cells infiltration were the essential characteristics of the disease. The inflammatory cells infiltration mediates renal fibrosis. Based on the differentiation of the microenvironment, describing the heterogeneity of DN may provide a new approach to explore the mechanisms of disease progression.

In this study, we propose that the glomerular microenvironment in DN exhibits significant immune and stromal heterogeneity, which is likely driven by specific key gene clusters and may influence the clinical progression of the disease. To systematically characterize this heterogeneity, consensus clustering was performed based on the abundance of cellular infiltration within the DN microenvironment to delineate sample-level microenvironmental heterogeneity. Subsequently, key pathogenic genes associated with tissue phenotype and microenvironmental heterogeneity were screened, and a scoring model was constructed using the principal components of these genes. This scoring model enables quantitative description of the microenvironment in DN samples and characterization of disease progression. Among the 15 key genes identified, ABCC9 was selected as the target gene for in-depth functional validation based on the following three considerations: First, ABCC9 demonstrates highly significant expression differences across distinct microenvironment subtypes and strongly correlates with the PCA score representing heterogeneity, indicating its close association with phenotypic variation. Second, compared to well-studied classical genes in DN fibrosis such as FN1 and TGFB2, the role of ABCC9 (an ATP-binding cassette transporter) in glomerular lesions of DN remains unclear, offering greater exploratory value. Finally, it has been reported to participate in cellular stress and inflammatory responses in other tissues, providing a biological rationale for its potential role in the DN microenvironment. Subsequent functional experiments demonstrated that ABCC9 can mitigate high glucose-induced increases in apoptosis and enhanced migration in human mesangial cells.

## 2 Methods and materials

### 2.1 Data download and bioinformatics analysis

We downloaded RNA sequencing datasets of DN tissue and normal kidney tissue (GSE142025 and GSE96804) from the Gene Expression Omnibus (GEO) database. GSE142025 included 21 advanced DN samples, six early DN samples, and nine normal samples. GSE96804 consisted of 41 DN glomeruli samples and 20 control samples. We used GSE96804 as the training cohort and GSE142025 as the validation cohort. The RNA sequencing data was transformed into immune cell and stromal cell infiltration data using the xCell algorithm. The immune score and stromal score of DN and normal samples were calculated using the ESTIMATE algorithm. To systematically characterize the microenvironmental heterogeneity of DN samples, unsupervised classification was performed using consensus clustering based on Ward’s method. By iteratively running clustering across a predefined range of cluster numbers K (K = 2–9), consensus matrices were generated for each K, and their corresponding cumulative distribution function (CDF) curves were calculated. The number of sample classifications was determined based on the change trend in the cumulative distribution function within the consensus index range. Differentially expressed genes (DEGs) between different phenotypes were screened. Genes that met the criteria of |log2FC| > 1 and false discovery rate (FDR) < 0.05 were retained. Enrichment analysis of DEGs was performed using the R package clusterProfiler based on the Gene Ontology (GO) and Kyoto Encyclopedia of Genes and Genomes (KEGG) databases, with the human genome as the background. KEGG pathways and GO terms were retained under the condition of q < 0.05. A two-dimensional plane was constructed using principal component 1 and principal component 2 to represent the phenotypes of the samples via principal component analysis (PCA). PCA was performed on standardized data. Key genes were identified through a multi-step cross-validation strategy. DEGs between normal and DN tissues were first identified from two cohorts using thresholds of FDR < 0.05 and |logFC| > 1. Second, Gene Set Variation Analysis (GSVA) was performed using 17 predefined gene sets related to diabetic complications, renal fibrosis, and immune inflammation, and genes significantly enriched (FDR < 0.05) across datasets were extracted. Finally, the common DEGs and GSVA-significant genes were integrated by Venn diagram intersection.

### 2.2 Cell culture

Human mesangial cell line (HMCs) for cell culture was purchased from Beijing Beina Chuanglian Biotechnology Institute. The cells were cultured in RPMI1640 medium containing 10 % FBS at 37 °C in a 5 % CO2 cell culture incubator. The medium was changed every 1–2 days based on the cell growth status, and subsequent experiments were conducted when the cells were in the logarithmic growth phase.

### 2.3 Cell transfection and grouping

Cells were divided into five groups according to a random number table: negative control group (NC group, 5.6 mmol/L glucose), knockdown control group (shNC, 5.6 mmol/L glucose), high glucose group + shNC (HG group, 30 mmol/L glucose), ABCC9 knockdown group (shAB group, 5.6 mmol/L glucose), and shAB + HG group (shAB + HG group, 30 mmol/L glucose). HMCs were seeded in a 6-well plate and when the cells reached 80 %–90 % confluence, shRNA was transfected into the well-growing mesangial cells. After 6 h of transfection, the culture medium was changed according to different conditions. After 48 h, the cells were used for subsequent experiments.

### 2.4 Transwell

Transfection was completed by following the above steps, cell counts were performed, and fine-tuned with serum-free medium. The cell suspension was added to the upper chamber, with a controlled cell number of 20,000, and 200 μL of medium containing serum was added to the lower chamber. The cells were fixed and stained. The experimental group and the negative control group were repeated three times, and five fields were randomly selected from each chamber to count the number of cells in each field.

### 2.5 qPCR

qPCR was used to detect ABCC9 mRNA expression. Total RNA was extracted using the Trizol method, and the RNA concentration and purity were measured. cDNA was synthesized using a reverse transcription kit according to the manufacturer’s instructions. Primer sequences used for real-time PCR were: ABCC9, forward 5′-CCG​TGT​CTC​TTC​TAT​TAT​GGA​TGC​AGG-3′ reverse 5′-CTA​CTT​GTT​GGT​CAT​CAC​CAA​AGT​GGA​AAA​G-3′. 1 µL of cDNA product was used for qPCR amplification. The amplification conditions were as follows: pre-denaturation at 95 °C for 30 s, 95 °C for 5 s, 55 °C for 30 s, 72 °C for 30 s, for 45 cycles; melting curve analysis: 95 °C for 5 s, 65 °C for 60 s, 95 °C for 1 s. The relative expression levels of each index were calculated using the 2^−ΔΔCt^ method.

### 2.6 Western blot

Western blot was used to detect protein expression. The intervened cells were collected in a 2 mL centrifuge tube, washed three times with PBS after centrifugation, and then added RIPA lysis buffer and broad-spectrum protease inhibitor for complete lysis. The supernatant was taken after centrifugation at 4 °C, 12,000*g* for 10 min, and the loading buffer was added and boiled for 10 min. 10 µL of protein sample was subjected to sodium dodecyl sulfate-polyacrylamide gel electrophoresis, and after transfer, 5 % skim milk was added and incubated for 2 h. After washing with TBST, ABCC9 primary antibody was added and incubated overnight at 4 °C. After washing with TBST for 10 min, 3 times, the secondary antibody (1:4,000) was added and incubated at room temperature for 1 h. After washing with TBST, the protein was visualized using chemiluminescence, and the results were detected using a scanner. ImageJ software was used to analyze the grayscale values of the proteins, with β-actin as the internal reference, to calculate the relative expression levels of the proteins. The experiment was repeated three times, and the grayscale values of the target proteins in different groups were compared.

### 2.7 Apoptosis detection

Cells were collected, centrifuged at 1,000 r/min for 5 min, and the culture medium was discarded. The cells were washed once with 3 mL PBS. After centrifugation, the PBS was removed, and the cells were fixed with pre-cooled 70 % ethanol at 4 °C for 1–2 h. After centrifugation, the fixing solution was discarded, and the cells were resuspended in 3 mL PBS for 5 min. The cells were filtered through a 400-mesh sieve, centrifuged at 500–1,000 r/min for 5 min, and the PBS was discarded. The cells were stained with 1 mL PI staining solution at 4 °C in the dark for 30 min. Flow cytometry analysis was performed within 1 h.

### 2.8 Statistical analysis

All quantitative experimental data in this study were derived from at least three independent replicate experiments, with results presented as mean ± standard deviation. Between-group comparisons for qPCR, Western blot, and cellular functional (migration/apoptosis) assays were performed using two-tailed *t*-tests or one-way ANOVA, with the significance threshold set at *P* < 0.05. For the integrated analysis of transcriptomic data, to eliminate batch effects between different datasets (GSE142025 and GSE96804), all expression data were corrected using the ComBat algorithm prior to merging, ensuring the reliability of cross-cohort analytical results. The study used Spearman’s test for correlation analysis. The diagnostic performance was evaluated using the receiver operating characteristic curve (ROC). This study did not involve any intervention on animals or humans and therefore did not require Ethical Committee review.

## 3 Results

### 3.1 Consensus clustering based on the microenvironment of DN samples

**Figure 1:**
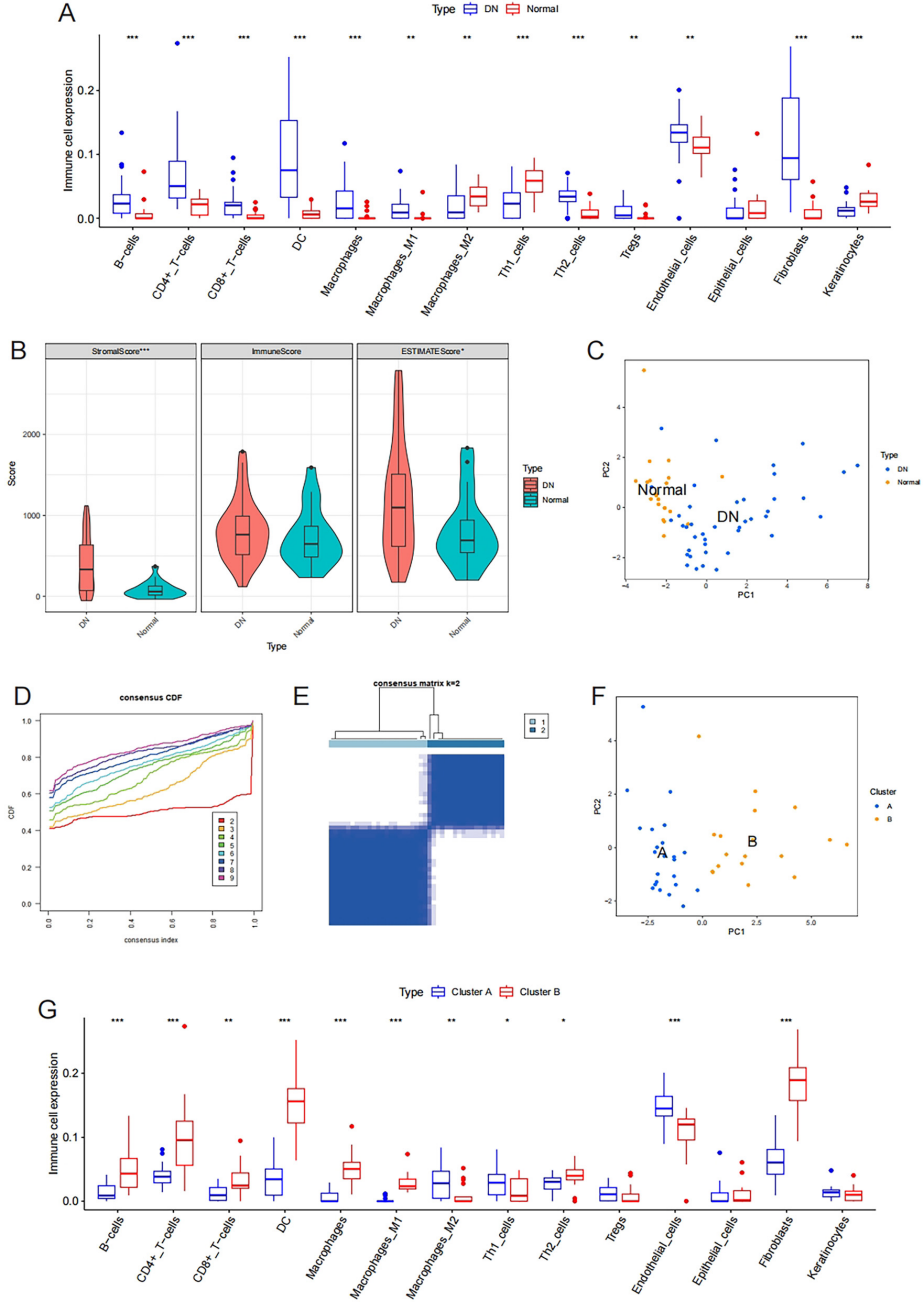
Consensus clustering based on the microenvironment of diabetic nephropathy (DN) samples. (A) The differences of immune cells and stromal cells infiltration between normal and DN samples. (B) The differences of stromal score, immune score and ESTIMATE score between normal and DN samples. (C) Principal component analysis of DN and normal samples based on cell infiltration in microenvironment. (D) Cumulative distribution function (CDF) of consensus clustering based on cell infiltration in DN microenvironment. (E) Consensus matrix of consensus clustering. (F) Principal component analysis of DN cluster A and cluster B samples based on cell infiltration in microenvironment. (G) The differences of immune cells and stromal cells infiltration between DN cluster A and cluster B samples.

Compared to normal glomeruli tissue samples, most immune cell subtypes and stromal cell subtypes (fibroblasts and endothelial cells) were upregulated in DN samples, as shown in Figure 1A. The stromal score and ESTIMATE score of DN samples were both higher than those of normal samples, as shown in Figure 1B. Principal component analysis was performed on all samples based on immune cell subtypes and stromal cell subtypes. In the coordinate system formed by principal component 1 and principal component 2, DN samples and normal samples were enriched in different regions, with lower principal component 1 in normal samples (Figure 1C). The microenvironment composed of immune cell subtypes and stromal cell subtypes is one of the essential characteristics of DN and can significantly distinguish it from normal samples. Consensus clustering was performed to stratify the samples and describe the heterogeneity of DN sample microenvironments. When K = 2, the cumulative distribution function showed a relatively flat trend within the range of consensus index from 0.1 to 0.9, as shown in Figure 1D. When K = 2, the consensus stromal had clear boundaries, thus dividing DN samples into two types, as shown in Figure 1E. Cluster A contained 23 samples, while cluster B contained 18 samples. Principal component analysis based on immune cell subtypes and stromal cell subtypes showed that cluster A and cluster B were distributed in different regions with good discrimination. Principal component 1 of cluster B was significantly higher than that of cluster A, as shown in Figure 1F. There were expression differences of immune cell subtypes and stromal cell subtypes between the two clusters, as shown in Figure 1G. The results of consensus clustering were reliable and accurate, and cluster A and cluster B exhibited distinct microenvironment phenotypes.

### 3.2 DEGs screening and enrichment analysis

**Figure 2:**
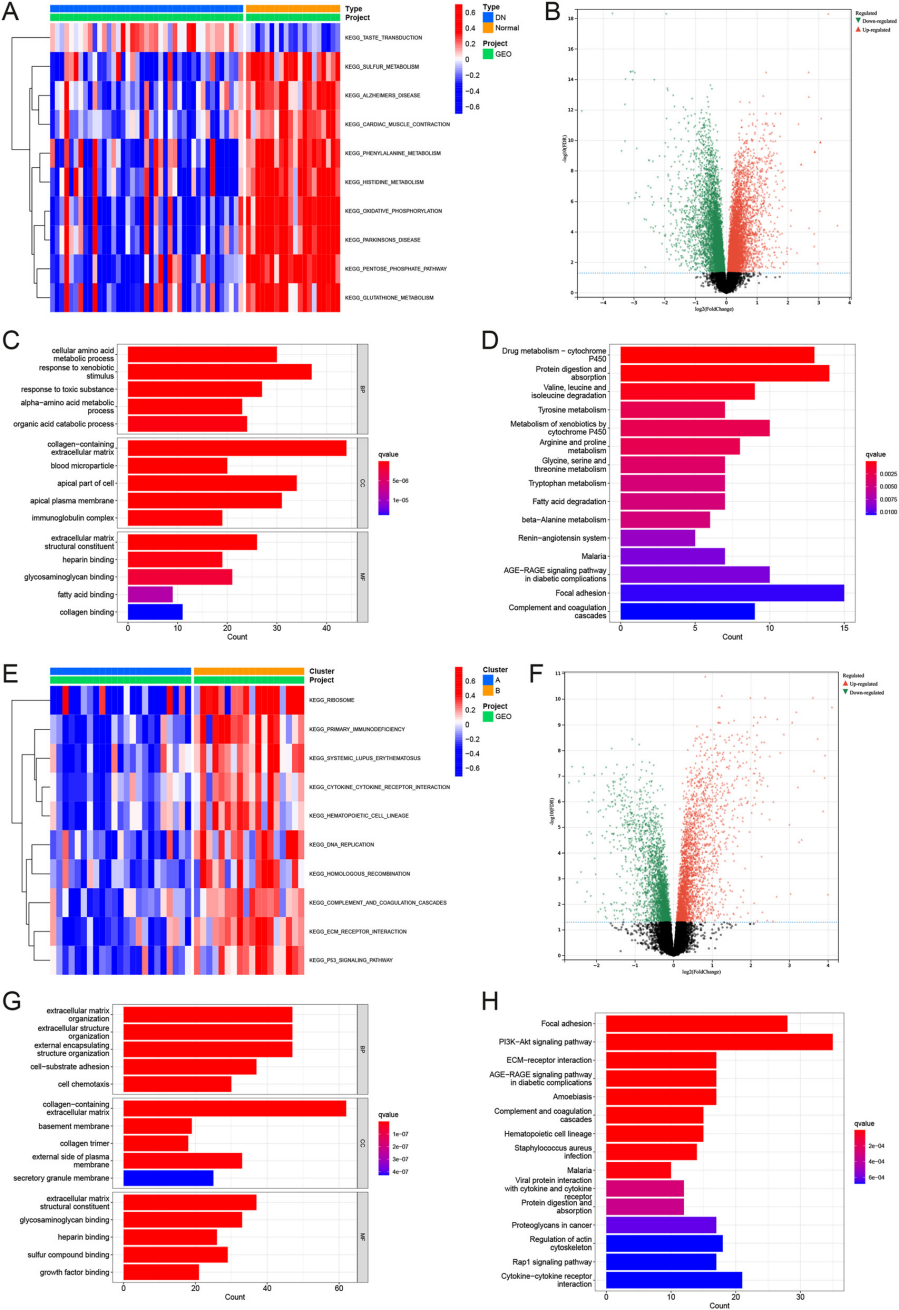
Differentially expressed gene (DEGs) screening and enrichment analysis. (A) Gene set variation analysis (GSVA) between DN and normal samples. (B) DEGs between DN and normal samples. (C) GO enrichment analysis of DEGs between DN and normal samples. (D) KEGG enrichment analysis of DEGs between DN and normal samples. (E) Gene set variation analysis (GSVA) between DN cluster A and cluster B samples. (F) DEGs between DN cluster A and cluster B samples. (G) GO enrichment analysis of DEGs between DN cluster A and cluster B samples. (H) KEGG enrichment analysis of DEGs between DN cluster A and cluster B samples.

To further investigate the mechanisms underlying phenotypic differentiation and elucidate the pathogenesis and progression of DN, DEGs between different phenotypes were identified and subjected to enrichment analysis. Gene set variation analysis (GSVA) showed that compared with normal samples, the gene sets of oxidative phosphorylation, pentose phosphate pathway and glutathione metabolism were highly expressed in DN samples, which may be potential pathogenic mechanisms of DN, as shown in Figure 2A. Compared with normal samples, 338 genes were highly expressed and 306 genes were down-expressed in DN, as shown in Figure 2B and Table S1. BP entries in which DEGs were significantly enriched between normal samples and DN samples included cellular amino acid metabolic process,alpha-amino acid metabolic process and organic acid catabolic process. CC items in which DEGs were significantly enriched between normal samples and DN samples included collagen-containing extracellular stromal, apical part of cell and immunoglobulin complex. DEGs between normal sample and sample DN significant enrichment of MF items including extracellular stromal structural constituent, glycosaminoglycan binding and fatty acid binding (Figure 2C). KEGG pathways in which DEGs are significantly enriched between normal and DN samples include AGE-RAGE signaling pathway in diabetic complications, renin-angiotensin system and amino acid metabolism (Figure 2D). GSVA showed that compared with DN cluster A, cytokine receptor interaction, ECM receptor interaction and P53 signaling pathway Gene sets of these pathways are highly expressed in DN cluster B (Figure 2E). Compared with DN cluster A, 187 genes had high expression in cluster B and 277 genes had low expression in cluster B (Figure 2F–Table S2). BP entries with significantly enriched DEGs between DN cluster A and DN cluster B include extracellular stromal organization, extracellular structure organization and cell chemotaxis. The CC items with significantly enriched DEGs between DN cluster A and DN cluster B samples included collagen-containing extracellular stromal, basement membrane and collagen trimer. MF entries with significant enrichment of DEGs between DN cluster A and DN cluster B include extracellular stromal structural constituent, glycosaminoglycan binding, growth factor binding (Figure 2G). KEGG pathways where DEGs are significantly enriched between DN cluster A and DN cluster B samples include the PI3K-Akt signaling pathway. ECM-receptor interaction and AGE-RAGE signaling pathway in diabetic complications (Figure 2H).

### 3.3 Key gene selection and establishment of scoring model

**Figure 3:**
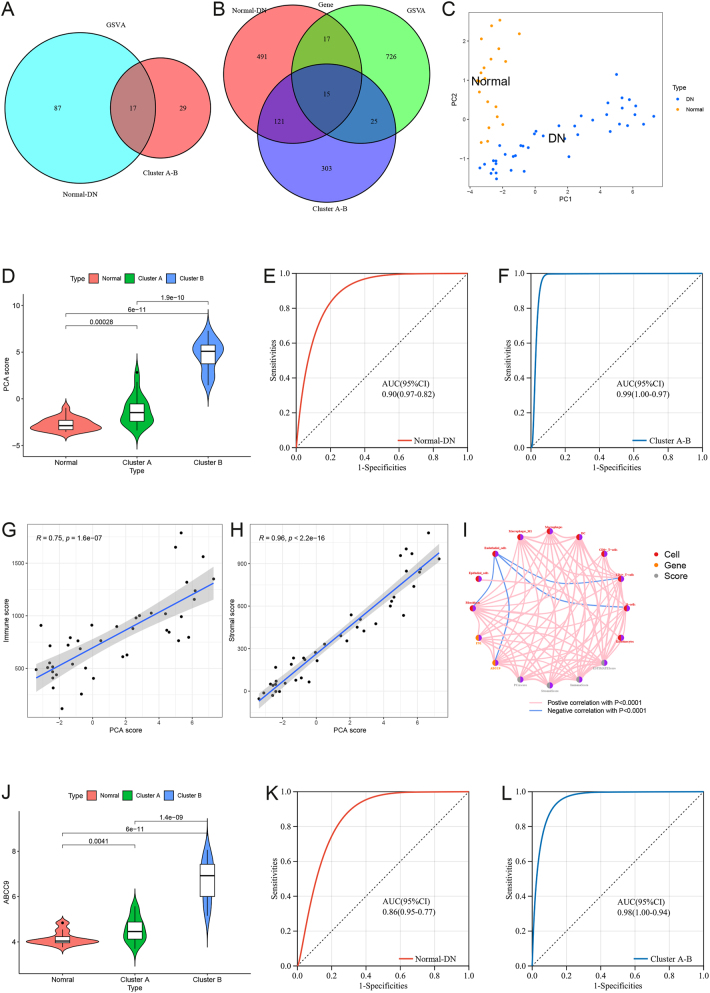
Key gene selection and establishment of principal component analysis (PCA) score. (A) The common variation gene sets between different phenotypes and different clusters. (B) The common genes between variation gene sets and differentially expressed gene sets. (C) Principal component analysis of DN and normal samples based on key genes. (D) The difference of PCA score between normal samples, DN cluster A samples, and DN cluster B samples. (E) Receiver operating characteristic (ROC) curve of PCA score between normal and DN samples. (F) ROC curve of PCA score between DN cluster A and B samples. (G) The correlation between immune score and PCA score. (H) The correlation between stromal score and PCA score. (I) The correlation between scores, genes and cells. (J) The difference of ABCC9 between normal samples, DN cluster A samples, and DN cluster B samples. (K) ROC curve of ABCC9 between normal and DN samples. (L) ROC curve of ABCC9 between DN cluster A and B samples.

In both normal and DN samples, there are 17 common mutated genes between DN cluster A and cluster B, as shown in Figure 3A. These 17 gene sets consist of 783 genes (Table S3). There are 15 genes in common between the GSVA gene set, the DEGs set between normal and DN samples, and the DEGs set between DN cluster A and cluster B, namely FN1, EGR1, TPM1, CCND2, COL1A2, TGFB2, COL6A3, ITGA11, ABCC9, THBS2, TNC, COL3A1, C7, C1QC, and ITGB6 (Figure 3B). We define these 15 genes as key pathogenic genes. Principal component analysis was performed based on these key pathogenic genes. Normal samples and DN samples were enriched in different regions, and this distribution trend was similar to the results of PCA based on cell subtypes (Figure 3C). PC1 of DN was higher than that of normal samples. We defined PC1 of the 15 key genes as PCA score. PCA score was the lowest in normal samples, followed by DN cluster A, and the highest in DN cluster B (Figure 3D). The ROC curve showed that PCA score can effectively distinguish normal samples from DN samples (AUC = 0.90, 95 % CI [0.82–0.97], Figure 3E), and can effectively distinguish DN cluster A samples from DN cluster B samples (AUC = 0.99, 95 % CI [0.97–1.00], Figure 3F). PCA score was positively correlated with immune score (r = 0.75, *P* = 1.6e-7, Figure 3G). PCA score was positively correlated with stromal score (r = 0.96, *P* < 2.2e-16, Figure 3H). Among the 15 key pathogenic genes, ABCC9 was selected as the target gene for further investigation. Figure 3I shows the extensive positive correlation between PCA score, ABCC9, immune cells, and stromal cells. ABCC9 had the lowest expression level in normal samples and the highest expression level in DN cluster B (Figure 3J). The ROC curve showed that ABCC9 can effectively distinguish normal samples from DN samples (AUC = 0.86, 95 % CI [0.77–0.95], Figure 3K), and can effectively distinguish DN cluster A samples from DN cluster B samples (AUC = 0.98, 95 % CI [0.94–1.00], Figure 3L).

### 3.4 External cohort validation

**Figure 4:**
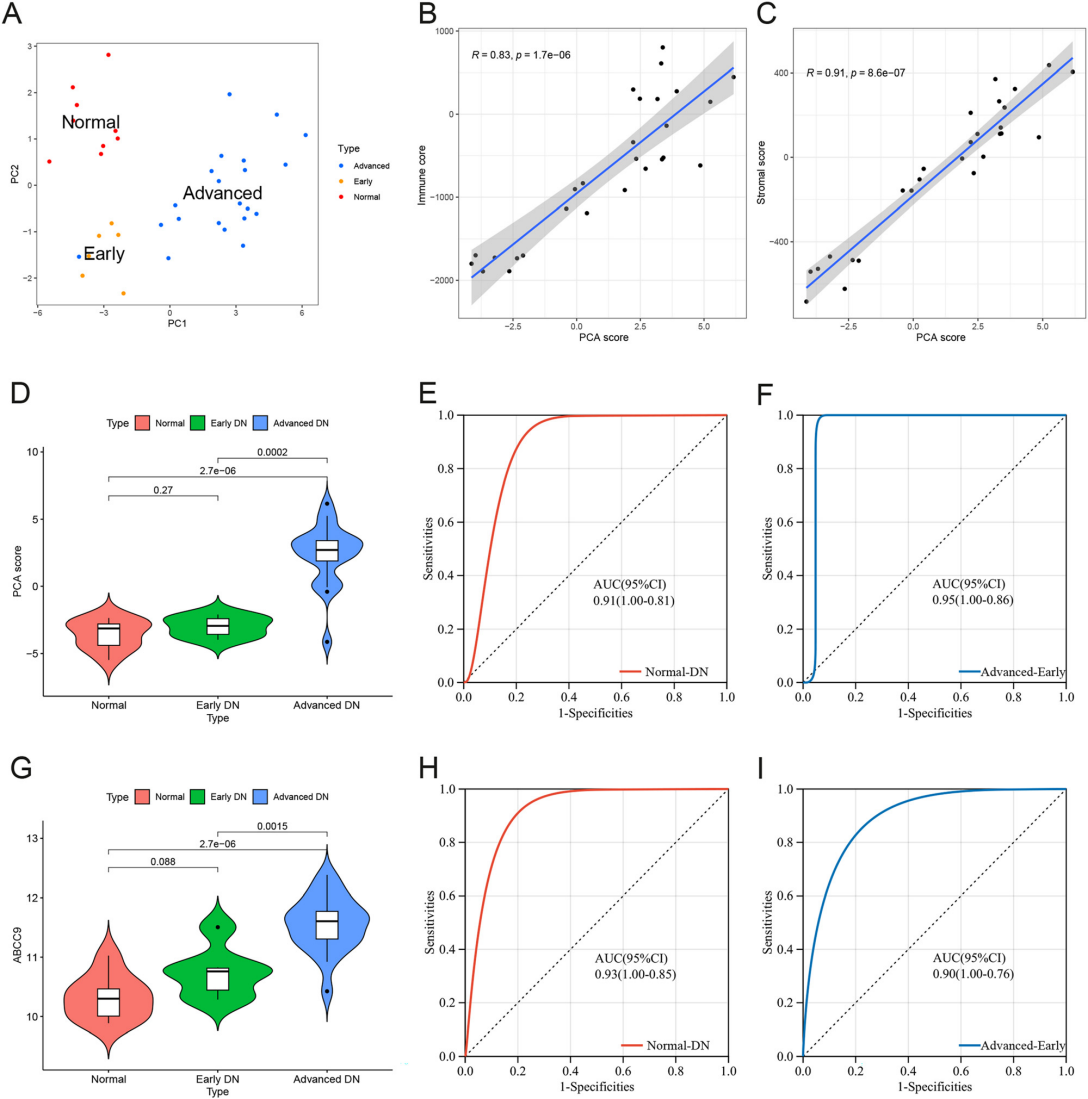
External cohort validation. (A) Principal component analysis (PCA) of DN and normal samples based on key genes in the validation cohort. (B) The correlation between immune score and PCA score. (C) The correlation between stromal score and PCA score. (D) The difference of PCA score between normal samples, early DN samples, and advanced DN samples. (E) Receiver operating characteristic (ROC) curve of PCA score between normal and DN samples. (F) ROC curve of PCA score between early and advanced DN samples. (G) The difference of ABCC9 between normal samples, early DN samples, and advanced DN samples. (H) ROC curve of ABCC9 between normal and DN samples. (I) ROC curve of ABCC9 between early and advanced DN samples.

To confirm the accuracy of the PCA scoring model and the role of ABCC9 in DN, we performed external cohort validation. Based on the expression levels of 15 key pathogenic genes, we conducted principal component analysis on the samples from the GSE142025 cohort. Normal samples, early DN samples, and advanced DN samples were distributed in different regions (Figure 4A). In the external validation cohort, PCA score was positively correlated with immune scoring (r = 0.83, *P* = 1.7e-6, Figure 4B). PCA score was also positively correlated with stromal scoring (r = 0.91, *P* = 8.6e-07, Figure 4C). In the external validation cohort, the PCA score of advanced DN samples was higher than that of normal samples and early DN samples (Figure 4D). The ROC curve showed that PCA score could effectively distinguish normal samples from DN samples (AUC = 0.91, 95 % CI [0.81–1.00], Figure 4E), as well as DN cluster A samples from DN cluster B samples (AUC = 0.95, 95 % CI [0.86–1.00], Figure 4F). In the external validation cohort, the expression level of ABCC9 was highest in advanced DN samples, higher than that in normal samples and early DN samples (Figure 4G). The ROC curve showed that PCA score could effectively distinguish normal samples from DN samples (AUC = 0.93, 95 % CI [0.85–1.00], Figure 4H), as well as early DN samples from advanced DN samples (AUC = 0.90, 95 % CI [0.76–1.00], Figure 4I).

### 3.5 Effects of ABCC9 on mesangial cells

**Figure 5:**
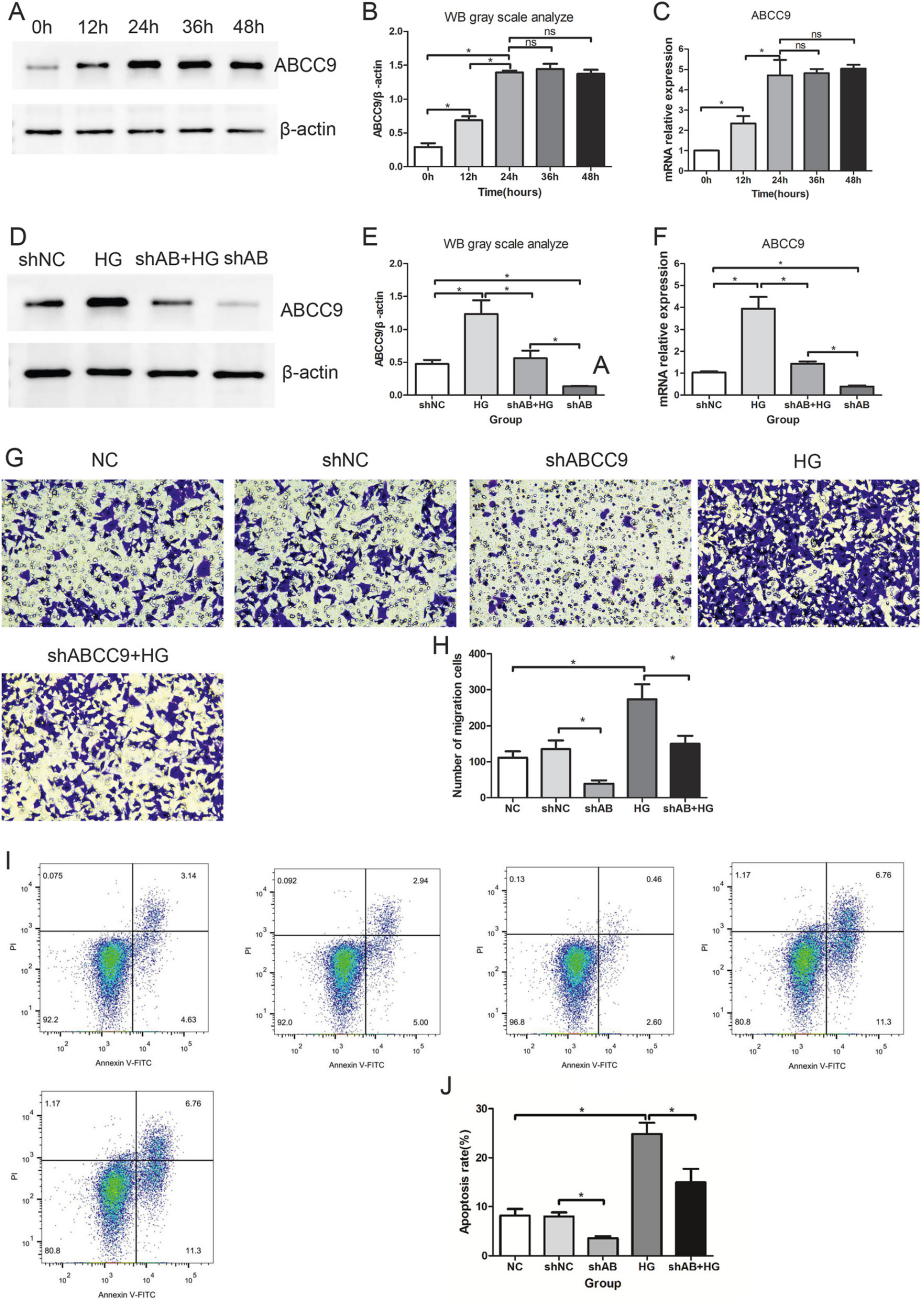
Effects of ABCC9 on mesangial cells. (A) The ABCC9 protein expression after high glucose stimulation for different time. The gel was cut. Both ABCC9 and β-actin were obtained from the same gel. See Supplementary Information Figure S1 for the original image. (B) Gray scale analyze of ABCC9/β-actin after high glucose stimulation for different time. (C) The ABCC9 mRNA expression after high glucose stimulation for different time. (D) The ABCC9 protein expression after ABCC9 knockdown and high glucose stimulation. The gel was cut. Both ABCC9 and β-actin were obtained from the same gel. See Supplementary Information Figure S2 for the original image. (E) Gray scale analyze of ABCC9/β-actin after ABCC9 knockdown and high glucose stimulation. (F) The ABCC9 mRNA expression after ABCC9 knockdown and high glucose stimulation. (G and H) Number of migrating cells after ABCC9 knockdown and high glucose stimulation. (I and J) Number of apoptotic cells after ABCC9 knockdown and high glucose stimulation.

After high-glucose treatment of mesangial cells, the expression level of ABCC9 protein increased over time. ABCC9 protein expression in mesangial cells was significantly higher at 24 h of high-glucose treatment compared to 0 h and 12 h (Figure 5A and ureB and Figure S1). The ABCC9 mRNA expression in mesangial cells also increased over time after high-glucose treatment. The ABCC9 mRNA expression in mesangial cells was significantly higher at 24 h of high-glucose treatment compared to 0 h and 12 h. After more than 24 h of high-glucose treatment, there were no significant changes in the ABCC9 protein and mRNA expression (Figure 5C). After ABCC9 knockout, both the protein (Figure 5D and ureE and Figure S2) and mRNA (Figure 5F) expression levels of ABCC9 were lower in the shAB group compared to the shNC group. After high-glucose stimulation, the protein and mRNA expression levels of ABCC9 in the shAB + HG group were higher than those in the shAB group but lower than those in the HG group (Figure 5D–F). Compared to the shNC group, the HG group showed an increase in the number of migrated cells (Figure 5G and H). Compared to the shNC group, the shAB group showed a decrease in the number of migrated cells (Figure 5G and H). Compared to the HG group, the shAB + HG group showed a decrease in the number of migrated cells (Figure 5G and H). Compared to the shNC group, the HG group showed an increase in the number of apoptotic cells (Figure 5I and J). Compared to the shNC group, the shAB group showed a decrease in the number of apoptotic cells (Figure 5I and J). Compared to the HG group, the shAB + HG group showed a decrease in the number of apoptotic cells (Figure 5I and J).

## 4 Discussion

We found that the majority of immune cell subtypes and stromal cell subtypes (fibroblasts and endothelial cells) were upregulated in DN samples. Principal component analysis based on cell subtypes showed good discrimination between DN and normal samples. Immune and stromal cell subtypes can be used to distinguish between normal tissue phenotypes and pathological tissue phenotypes. Our analysis suggests that changes in the microenvironment are a crucial step in the development of DN. The immune and stromal microenvironment is one of the essential characteristics of DN and serves as a scale to describe disease heterogeneity and clinical phenotypes. It is an important factor that cannot be ignored in exploring disease mechanisms and discovering therapeutic targets. We performed consensus clustering of DN samples based on immune cell subtypes and stromal cell subtypes, hoping that this classification could describe the heterogeneity of the microenvironment in DN samples. We divided DN samples into two clusters. DN cluster A is characterized by high infiltration of immune cell subtypes and high infiltration of fibroblasts, while DN cluster B is the opposite.

In the subtype-based principal component analysis, DN cluster A and DN cluster B showed good discrimination. The DN samples in these two clusters exhibited distinct microenvironmental characteristics. Compared to normal samples, the DN samples had higher levels of M1 macrophages and fibroblasts, and lower levels of M2 macrophages. Compared to DN cluster A, DN cluster B had higher levels of M1 macrophages and fibroblasts, and lower levels of M2 macrophages. This is consistent with the pathological process of DN. Immune cells may act as a “bridge” between hyperglycemia and renal fibrosis in the progression of DN [22], 23]. Fibrosis is a compensatory response to injury under normal conditions [24], 25]. During the self-repair process of damaged tissues, immune cells can synthesize various pro-fibrotic cytokines, and the immune microenvironment remains in a sustained high-reactive state, which becomes the central driving factor for progressive renal fibrosis [26], [27], [28]. Immune cells associated with renal fibrosis include macrophages, neutrophils, lymphocytes, etc. [29], [30], [31]. Kidneys of DN patients are often accompanied by infiltration of macrophages. M1 and M2 macrophages maintain a balance in the process of renal fibrosis (fibrogenic mediators) and anti-fibrosis (fibrolytic enzymes). If the kidneys are exposed to a high-glucose environment and hemodynamic disorders for a long time, the abnormally accumulated macrophages in the kidneys often become dysfunctional, leading to thickening of the glomeruli basement membrane, proliferation of mesangial cells, and interstitial fibrosis, accelerating the progression of DN. The enrichment of neutrophils and lymphocytes in renal tissues also promotes the development of renal fibrosis [32], [33], [34]. Changes in the microenvironment are present throughout the various stages of DN occurrence and progression, and they are the driving factors and direct manifestations of DN progression [35]. The consensus clustering based on cell subtypes accurately differentiated DN samples with different microenvironmental characteristics, providing a better description of the heterogeneity of DN samples and the developmental process of DN.

In order to further elucidate the mechanisms of phenotypic differentiation and the heterogeneity of the microenvironment in DN samples, we performed GSVA and enrichment analysis. ECM-receptor interaction and oxidative phosphorylation may be potential mechanisms underlying the progression of DN. We intersected the DEGs and variant genes between different phenotypes, resulting in a total of 15 common genes. Based on the expression levels of these 15 genes, we conducted PCA on all samples. DN samples and normal samples were enriched in different regions, and this distribution trend was consistent with the distribution trend based on immune cell subtypes and stromal cell subtypes in the PCA. We defined the PC1 of these 15 genes as the PCA score. The PCA score gradually increased in normal samples, DN cluster A, and DN cluster B. We also found that PCA score had good diagnostic performance for DN samples. PCA score showed correlations with immune score and stromal score. PCA score can reflect the microenvironment of DN, describe the heterogeneity of samples, and reflect the severity of the disease. We validated the accuracy and reliability of PCA score in an external cohort. In the PCA based on the 15 key pathogenic genes, normal samples, early DN samples, and late DN samples were distributed in different regions, which was consistent with the distribution trend of different phenotypes in the previous PCA. In the external validation cohort, PCA score accurately reflected the immune cells and stromal cells infiltration in the microenvironment. PCA score had good diagnostic performance for DN samples and normal samples, as well as for early DN samples and late DN samples.

Among the 15 key genes, we selected ABCC9 as the target gene for further study to elucidate its effects on the biological behavior of glomeruli mesangial cells. ABCC9 was upregulated in DN samples and had good diagnostic performance for DN. The expression level of ABCC9 could effectively distinguish early DN samples from late DN samples. ABCC9 showed extensive correlations with microenvironmental cell subtypes. We found that in glomeruli mesangial cells, the expression of ABCC9 increased with prolonged exposure to high glucose stimulation. ABCC9 could counteract the increase in apoptosis and migration of glomeruli mesangial cells induced by high glucose. Previous studies have not investigated the role of the ABCC9 gene in DN. The ABCC9 gene is an “insomnia gene” that is evolutionarily ancient and exists in the heart, skeletal muscle, brain, and pancreas of mammals. It can determine the duration of sleep. The protein encoded by the ABCC9 gene is one of the components of potassium ion channels [36]. ABCC9 has been shown to promote hippocampal aging and sclerosis and is a potential therapeutic target [37], 38]. ABCC9 is involved in brain neurovascular development and neurovascular coupling [39]. ABCC9 has also been shown to participate in oxidative metabolism [40].

This study has several limitations that should be acknowledged. Firstly, both the discovery and validation cohorts were relatively small in size, which may affect the statistical power and generalizability of the findings. To address this, future studies should incorporate larger, multi-center cohorts or publicly available datasets with deeper clinical annotations. Secondly, the current work focused solely on diabetic nephropathy and did not explore whether similar microenvironmental heterogeneity and key genes, such as ABCC9, play roles in other diabetic complications, such as retinopathy, neuropathy, or vasculopathy. Extending this framework to other diabetes-related organ injuries represents an important future direction. Finally, while we demonstrated the functional relevance of ABCC9 in mesangial cells under high-glucose conditions, the precise molecular mechanisms through which ABCC9 influences apoptosis, migration, and potentially other fibro-inflammatory processes remain unclear. Further mechanistic studies, including identification of upstream regulators, downstream signaling pathways, and *in vivo* validation, are warranted to fully elucidate its pathogenic role in DN progression. Repurposing drugs and giving various vitamins as D as prophylactic with immuno-modulatory effect with positive impact on DN [41].

## 5 Conclusions

FN1, EGR1, TPM1, CCND2, COL1A2, TGFB2, COL6A3, ITGA11, ABCC9, THBS2, TNC, COL3A1, C7, C1QC, and ITGB6 are potential key pathogenic genes in DN. The PCA score constructed based on these genes can distinguish between normal tissue phenotype and DN phenotype, quantitatively describing the immune microenvironment and stromal microenvironment of DN, reflecting the progression of DN. Knocking out ABCC9 can counteract the increased apoptosis and migration of glomeruli mesangial cells induced by high glucose.

## Supplementary Material

Supplementary Material

Supplementary Material

Supplementary Material
